# Superstrong Carbon Nanotube Yarns by Developing Multiscale Bundle Structures on the Direct Spin‐Line without Post‐Treatment

**DOI:** 10.1002/advs.202204250

**Published:** 2022-11-20

**Authors:** Young Shik Cho, Jae Won Lee, Jaewook Kim, Yeonsu Jung, Seung Jae Yang, Chong Rae Park

**Affiliations:** ^1^ Department of Materials Science & Engineering and Research Institute of Advanced Materials Seoul National University Seoul 08826 Republic of Korea; ^2^ Composite Research Division Korea Institute of Materials Science (KIMS) Changwon 51508 Republic of Korea; ^3^ Department of Chemistry & Chemical Engineering Education and Research Center for Smart Energy and Materials Inha University Incheon 22212 Republic of Korea

**Keywords:** bioinspired, bundle engineering, carbon nanotube, superstrong, yarn

## Abstract

Super strong fibers, such as carbon or aramid fibers, have long been used as effective fillers for advanced composites. In this study, the highest tensile strength of 5.5 N tex^−1^ for carbon nanotube yarns (CNTYs) is achieved by controlling the micro‐textural structure through a facile and eco‐friendly bundle engineering process in direct spinning without any post‐treatment. Inspired by the strengthening mechanism of the hierarchical fibrillary structure of natural cellulose fiber, this study develops multiscale bundle structures in CNTYs whereby secondary bundles, ≈200 nm in thickness, evolve from the assembly of elementary bundles, 30 nm in thickness, without any damage, which is a basic load‐bearing element in CNTY. The excellent mechanical performance of these CNTYs makes them promising substitutes for the benchmark, lightweight, and super strong commercial fibers used for energy‐saving structural materials. These findings address how the tensile strength of CNTY can be improved without additional post‐treatment in the spinning process if the development of the aforementioned secondary bundles and the corresponding orientations are properly engineered.

## Graphical abstract

This study focuses on improving the mechanical performance of carbon nanotube yarns (CNTYs) without any post‐treatment in direct spinning. Inspired by the strengthening mechanism of natural cellulose fiber, micro‐texture controlled CNTYs are produced with the highest ever tensile strength of 5.5 N tex^−1^ reported thus far, surpassing the value of 3.9 N tex^−1^ of the strongest carbon fiber, T1100G.

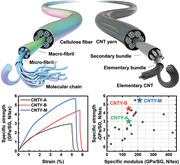

## 1 Introduction

Structural materials have been significant in developing some of the latest equipment in various fields, such as aerospace, automobile, and many other sectors. In particular, lightweight and super strong structural materials (e.g., carbon fibers and superfibers) for improving the efficiency of engines in industries, such as aircraft, will be indispensable for energy saving in the future. Furthermore, to advance to the dreamlike space elevator,^[^ ^1^ ^]^ the cable components for the elevator need to be lightweight and super strong to endure the weight of the entire system. Among super strong and lightweight materials, carbon nanotubes (CNTs) have been highlighted as the most promising material^[^ ^2^ ^]^ owing to their outstanding mechanical properties, which include a theoretical modulus and strength as high as 1 TPa and 77 GPa g^−1^ cm^3^ [= N tex^−1^],^[^ ^3^ ^]^ respectively. On the other hand, these excellent properties of CNTs could never be accomplished in fibers, as it is almost impossible to make infinitely long filamentous CNTs with currently available technologies. A CNT yarn (CNTY),^[^ ^4^ ^]^ a thread composed of CNTs at a micron‐sized length, aligned one‐dimensionally along the axis and laterally overlapped, has been suggested.

Several techniques have been adopted to produce CNTYs, including drawing from a CNT forest,^[^ ^5^ ^]^ direct spinning from a furnace, in which CNTs are growing^[^ ^6^, ^7^, ^8^ ^]^ and wet or dry‐jet‐wet spinning from a solution with the CNTs in a liquid crystalline phase.^[^ ^9^, ^10^, ^11^, ^12^, ^13^ ^]^ In conjunction with these techniques, a few additional processes, such as solvent densification, twisting, or both, have been assessed to enhance the tensile strength of as‐spun CNTYs.^[^ ^8^, ^14^ ^]^ On the other hand, the recently achieved best tensile strength was 5.5 N tex^−1^,^[^ ^15^ ^]^ which is virtually impossible without tedious, complicated, and environmentally unfriendly processes.^[^ ^8^, ^9^, ^10^, ^15^, ^16^, ^17^, ^18^ ^]^

**Figure 1:**
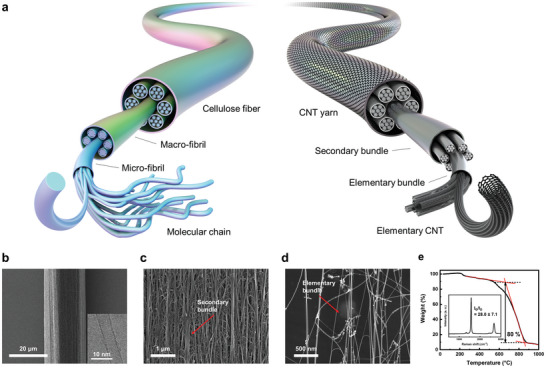
Similarity in the multiscale micro‐textural structures between natural cellulose fibers and CNTYs. a) Schematic diagram of natural cellulose fiber and CNTY. SEM images of b) CNTYs (inset: TEM micrograph of CNTs), c) secondary bundle from CNTY, and d) elementary bundle from CNT aerogel. TGA thermogram (inset: Raman spectra, *n* = 5) indicate that the synthesized CNTYs are mostly double‐walled carbon nanotubes.

Most natural fibers, such as cellulose fibers, consist of a four‐level hierarchy of polymer‐microfibril‐macrofibril‐fibers.^[^ ^19^ ^]^ Previous studies reported that the key mechanistic principle of such fiber is the development of a macrofibril while maintaining or strengthening the microfibril in crystallite form, which is the load‐bearing element.^[^ ^20^ ^]^ Because the hierarchical structure of CNTY is similar to that of natural cellulose fiber, as shown in **Figure** **1**a, it is necessary to focus on developing a macrofibril‐like bundle while maintaining a microfibril‐like bundle to improve the strength. Most post‐treatments were eventually conceived to produce a huge bundle, but the effect was not maximized because of the inevitable weakening of the underlying microfibril‐like structure.

Inspired by the strengthening mechanism and structural characteristics of the natural fiber, this paper reports an efficient method of engineering the multi‐level CNT bundle according to scale to improve the tensile strength of CNTY without using the toxic solvents used conventionally to develop larger bundles. Thus, superstrong CNTYs were fabricated by implementing a huge bundle while maintaining the intrinsic microstructure through a facile and eco‐friendly process, even without post‐treatment. Adopting an additional guide roller in the direct spinning process with mechanical conditioning was quite effective in increasing the bundle size, hence achieving the best tensile strength comparable to the currently reported highest performance of CNTYs produced by a process using a toxic, concentrated sulfuric acid. The specific strength of the CNTYs was improved to 5.5 N tex^−1^ by developing larger bundles of several hundred nm in size or more. This performance surpassed the highest tensile strength of ≈3.9 N tex^−1^ for the commercial carbon fiber, T1100. These findings will have significant implications in that the tensile strength of CNTYs can be enhanced further when multiscale bundles inspired by natural cellulose fiber, which plays a crucial role as the load‐bearing element, are properly engineered. Such an improvement is possible through a simple, eco‐friendly, and practically feasible process.

## 2 Results and Discussions

### 2.1 Bio‐Inspired Multiscale Micro‐Textural Structure of CNTY

Mimicking the concept of natural fibers has inspired a new approach to the hierarchy of CNTY. The balance between the stabilization energy^[^ ^21^ ^]^ of the CNTs assembled in a bundle and van der Waals interactions of CNTs results in a distinctive nanostructure, the CNT elementary bundle, which corresponds to the microfibril of natural fibers. The secondary bundle, a CNT assembly above the nano‐mechanical domain, was formed by van der Waals forces between the elementary bundles as macrofibril of natural fibers. Therefore, the CNT bundle was classified into two stages: CNT elementary bundle and the secondary bundle (Figure 1a). Because CNT nano‐assemblies have been reported to have high strength,^[^ ^22^ ^]^ the elementary bundle was regarded as the smallest load‐bearing unit, indicating that the final fracture of CNTY occurs due to slippage of the CNT elementary bundles in the secondary bundle. In this respect, a high‐strength CNTY can be produced by developing a larger secondary bundle. Hence, an engineering process specialized for aggregating each elementary and secondary bundle is required. In this study, the CNT assembly engineering was controlled during direct spinning without deformation of the elementary bundle to achieve high strength CNTY.

CNTY was synthesized using the direct spinning method with a diameter of 20 µm and a linear density of 0.114 tex [ = g km^−1^] composed of double‐walled CNTs (DWCNTs) with an outer diameter of ≈3 nm according to the transmission electron microscopy (TEM) images in Figure 1b and Figure S2a,b (Supporting Information). As shown in the scanning electron microscopy (SEM) images of CNTY, the fabricated CNTY consisted of secondary bundles (Figure 1c), hundreds of nm in diameter, and elementary bundles (Figure 1d and Figure S2c–f, Supporting Information), tens of nm in diameter. According to Figure S2d–f (Supporting Information), the elementary bundles were formed as well‐aligned CNT nano‐assemblies. These results suggest that the hierarchy observed in the natural cellulose fiber was also seen in CNTYs. According to the thermogravimetric analysis (TGA) curve in Figure 1e, a large mass reduction occurred after 600 °C, which is a typical sign of DWCNT.^[^ ^23^ ^]^ 79.2 wt% of CNTY was mostly composed of DWCNT while 11.2 and 9.2 wt% of the yarn were amorphous carbon and residual Fe, respectively. A high load‐bearing unit that is proportional to the amount of DWCNT contributes to the mechanical performance. According to Raman spectroscopy, the intensity ratio of the G‐band to D‐band (*I*_G_/*I*_D_) was ≈28.0 ± 7.1 (Figure 1e, *n* = 5), which is above 10, confirming that the elementary CNTs are highly crystalline and composed of two walls. Owing to the high quality of the elementary CNT, the mechanical properties of CNTY were predominantly affected by the multiscale bundle structures, such as elementary/secondary bundles.

### 2.2 The Multiscale Bundle Engineering without Post‐Treatment in CNTYs

**Figure 2:**
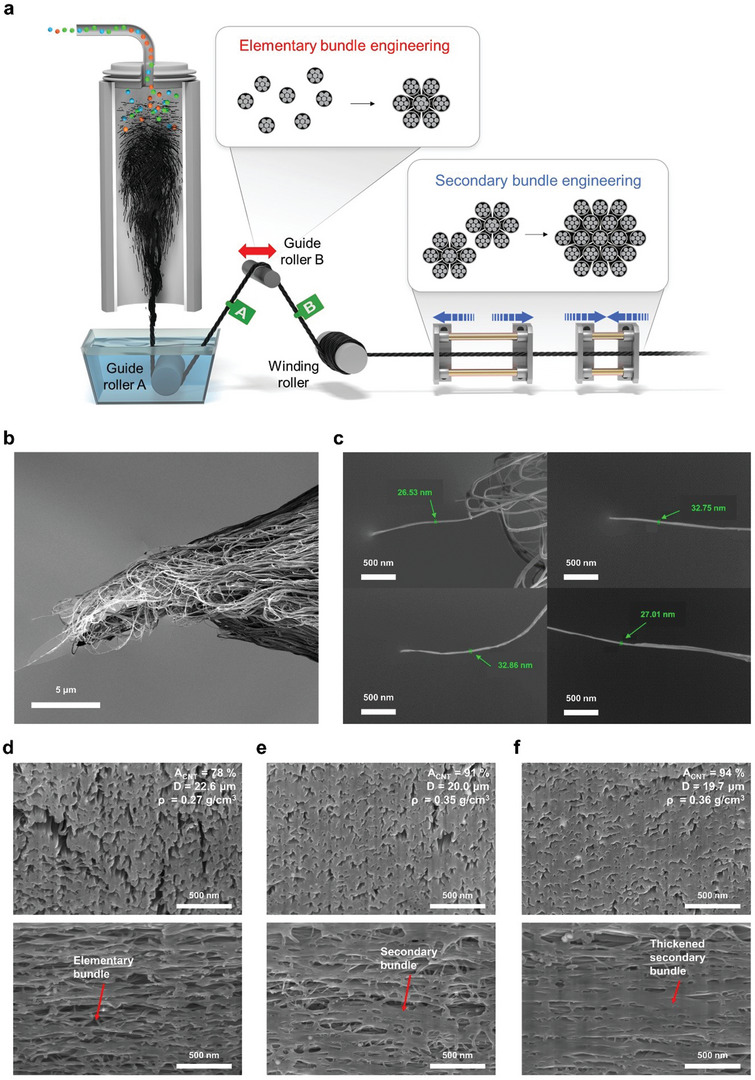
Development of the multiscale micro‐textural structure of CNTYs. a) Schematic diagram of the process for developing the multiscale micro‐textural structure on the direct spin‐line. SEM images of b) the fracture surface of CNTY‐M and c) elementary bundles with variable diameters at the end of the fracture surface. SEM images of FIB cut cross‐sections and radial sections of d) CNTY‐A, e) CNTY‐B, and f) CNTY‐M. Note the effective cross‐sectional area (*A*_CNT_), diameter (*D*), and volumetric density (*ρ*) of CNTYs in the upper right corner of the image.

The engineering of the multiscale bundle structures in CNTYs was proceeded through combination of direct spinning process and mechanical conditioning^[^ ^24^ ^]^ based on the load‐bearing mechanism of polymer fiber to control the assembly behavior of the CNT bundle. In the direct spinning process, as shown in **Figure** **2**a, the growing DWCNTs readily assemble into a CNT aerogel of elementary bundles in a furnace, which is then drawn through guide roller A in a water bath together with or without an additional guide roller B by a winding roller at a given rate. The CNTYs were fabricated in hundreds of meter scale homogeneously. (See the stress–strain curve of CNTY‐B measured at every 50 m interval an example, Figure S3, Supporting Information.) The mechanical conditioning of given cyclic strain was then adopted to winded CNTY. During this streamlined process, it is crucial to optimize the assembly behavior of the grown CNTs, which is the formation of a secondary bundle from an elementary bundle, because the characteristics of the CNT assembly play a critical role as the load‐bearing elements in the resulting yarns.

The synthesized as‐spun CNTYs were drawn at the winding rate of 6 m min^−1^ without any additional treatments. The CNTYs drawn through guide rollers A & B were denoted as CNTY‐B, while CNTY manufactured only through guide roller A was denoted as CNTY‐A. The mechanical conditioning of 2% cyclic strain was adopted to CNTY‐B and the mechanically conditioned CNTY was denoted as CNTY‐M. The CNTY‐M showed the highest strength due to the agglomeration of a secondary bundle, which is a freely movable element of CNTYs under dried state. (See Figure S4, Supporting Information for optimization of winding rate and cyclic strain.)

Fracture surface analysis of the CNTY‐M was performed to identify the unit of the load‐bearing element. A fracture surface of the CNTYs shown in Figure 2b depicts the appearance of a ruptured morphology, unlike previous reports that the CNTY fails through slippage between CNTs or bundles. An additional analysis was conducted at high magnification, with the results shown in Figure 2c. The CNT bundles with a diameter of ≈30 nm were found at most ends of the failure section. Because no CNT assembly structures with smaller diameters or individual CNTs were found at the fracture ends, the nano‐fibrillar bundle was identified as a primary structure formed by strong intertube interactions between CNTs. These results show that the load‐bearing element is not a simple bundle or individual CNT but an elementary bundle. CNTY failure occurs due to slippage between the elementary bundles, which appears as a rupture of the secondary bundle from a macroscopic point of view. The fracture surface of CNTYs shows that the elementary bundle can still maintain the structure and play a role in the macroscopic structure even after yarn formation. (See Figure S5a,b, Supporting Information for more evidence of microstructure mechanism.) Also, the assembly behavior of the bundle in the CNTY can be interpreted similarly to that of the fibril in the natural cellulose fiber.

The strength is closely related to the assembly behavior of CNT bundles. Hence, Raman spectroscopy was conducted before and during the tensile loading of CNTYs. The orthogonal electronic dispersion occurs when CNTs are in contact with each other and the G’ peaks shift to lower energies. This suggests that the G’ peak downshift reflects the inter‐nanotube contact area.^[^ ^25^ ^]^ Figure S5c (Supporting Information) shows the Raman G’ peak of the DWCNT yarns with Lorentzian fitting. The G’ peak shifted from 2674.4 cm^−1^ of CNTY‐A to 2666.7 cm^−1^ of CNTY‐B through self‐assembly of the elementary bundle and to 2664.5 cm^−1^ of CNTY‐M. By engineering the CNT bundles, dense packing was achieved from the initial static state on CNTY‐M. Focused ion beam (FIB) analysis of a cross‐section and radial section of CNTY‐A, CNTY‐B, and CNTY‐M was performed to identify the assembly behavior, as shown in Figure 2d–f, respectively. The tensile strength of the samples increased with the effective cross‐sectional area of the CNTYs, which is the proportion of the area occupied by the secondary bundles to the total area of a given image frame, from 78% to 91% and to 94% for CNTY‐A, CNTY‐B, and CNTY‐M, respectively. Furthermore, the volumetric density of CNTYs has increased from 0.27 to 0.35 and to 0.36 g cm^−3^ for CNTY‐A, CNTY‐B, and CNTY‐M, respectively. According to the theoretical density of DWCNT,^[^ ^26^ ^]^ dense arrangement of CNT bundles in CNTY‐A to CNTY‐M was successful, even considering that 11 wt% CNTY is an iron‐based catalyst. The FIB results matched the results from Raman spectroscopy, as a higher effective area with a lower amount of isolated elementary bundle led to a larger downshift of the G’ peak. Moreover, as bundle engineering progressed, the isolated regions (elementary bundles) in the radial section combined to a connected region (secondary bundle). Specifically, through engineering the elementary bundle, the isolated regions were clustered in the direction of increasing the number of ≈100 nm‐sized secondary bundles. In the case of secondary bundle engineering via mechanical conditioning, the area of the connected region, which is the size of the secondary bundle, increased to more than 200 nm. Most of the elementary bundles belong to larger secondary bundles that occupy almost all the cross‐sectional area of the CNTYs, while some elementary bundles adjoin the neighboring secondary bundles like tying molecules. Increasing the proportion of the secondary bundles in the yarn is critical for improving the tensile strength of CNTYs, suggesting that the imposed tensile deformation energy is mostly dissipated by the secondary bundles, but the final fracture ends up with slippage between the elementary bundles.

### 2.3 Mechanical Performance Evolution of CNTYs According to Micro‐Texture Development through Bundle Engineering

**Figure 3:**
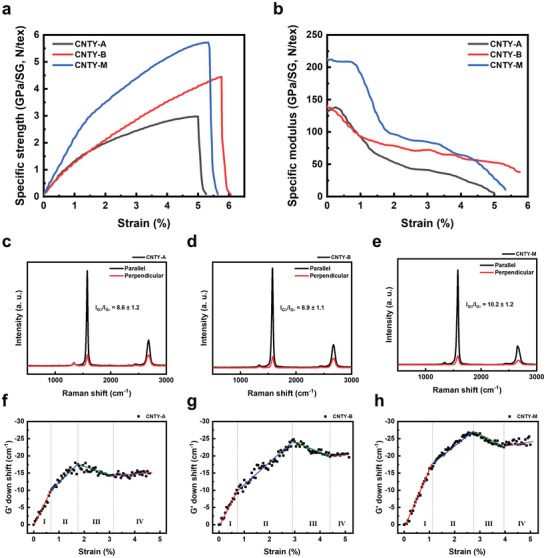
Mechanical properties and assembly behavior of multiscale bundles in CNTYs. a) Specific stress–strain curve and b) specific modulus–strain curve of CNTYs (*n* = 10). Polarized Raman spectra (top, *n* = 5) and down‐shift of the G’ peak positions (bottom, *n* = 3) of c,f) CNTY‐A, d,g) CNTY‐B, and e,h) CNTY‐M, showing the changes in the bundle size and corresponding orientations during the tensile deformation process.

The tensile performance of CNTYs was improved remarkably from 3.0 ± 0.2 N tex^−1^ in terms of the specific strength (140.6 ± 22.0 N tex^−1^ in terms of the specific modulus) for CNTY‐A (*n* = 10) to 4.5 ± 0.2 N tex^−1^ (141.1 ± 9.9 N tex^−1^) for CNTY‐B (*n* = 10) by drawing at second guide roller B and finally to 5.5 ± 0.2 N tex^−1^ (211.4 ± 14.6 N tex^−1^) for CNTY‐M (*n* = 10) via mechanical conditioning (**Figure** **3**a). (See the Supporting Information for synthesis condition^[^ ^8^, ^27^, ^28^ ^]^ and estimated aspect ratio^[^ ^16^ ^]^ of DWCNT for high strength CNTY.) The suggested bundle engineering can enhance the specific strength by 80% and the specific modulus by 50% from CNTY‐A to CNTY‐M. The resulting specific strength of CNTY‐B was higher than that (≈3.9 N tex^−1^) of the carbon fiber T1100, known as one of the strongest commercial fibers. Moreover, the specific strength of CNTY‐M has never been achieved by a direct spinning method without the use of a toxic solvent, such as concentrated sulfuric acid.

The specific modulus–strain curves of CNTYs (Figure 3b) show similar behavior in the overall tensile deformation, but there was a clear difference in curve intensity depending on the certain strain range. The specific modulus of the CNTYs decreased steeply initially until a certain point of strain and showed a gradual decline thereafter. The two‐mode decline of the modulus curve of CNTYs implies a hierarchical structure composed of elementary and secondary bundles.^[^ ^8^, ^29^ ^]^ At low strain, the load is transferred dominantly through the secondary bundle. Among the three CNTYs, only CNTY‐M shows a distinctly different modulus at strain under 1%, which means that the secondary bundle in the yarn developed through mechanical conditioning. On the other hand, as the strain increases, the load is transferred mainly to the elementary bundle and tying secondary bundles. In the 2–4% strain section, CNTY‐B and CNTY‐M showed similar modulus because both CNTYs underwent the development of the elementary bundle through self‐assembly during the spinning process. (Some of the differences were attributed to the densification of tying the secondary bundle during the mechanical conditioning process.) This difference in the modulus curves is solid evidence of the structural change after bundle engineering.

**Figure 4:**
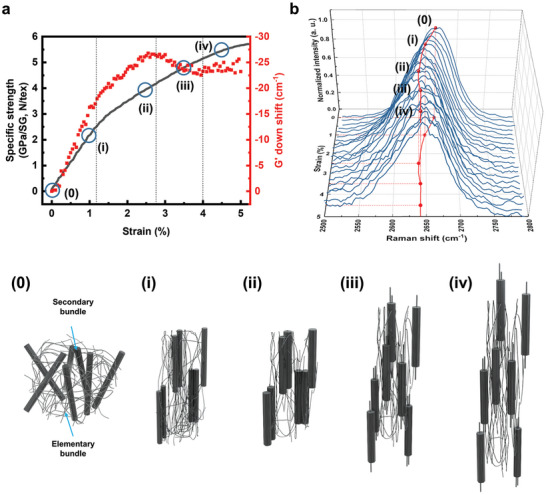
Suggested mechanism for the tensile fracture of CNTYs. a) Specific stress–strain curve with down‐shift of the G’ peak positions of CNTY‐M and b) in situ Raman spectra of CNTY‐M under strain applied. 0) Elementary bundles tying the secondary bundles (initial state), i) elastic realignment of the elementary and the secondary bundles oriented slightly off‐the‐yarn axis (Zone I), ii) reorientation of the misoriented tying bundles to adjacent secondary bundles (Zone II), iii) breakage of the tying bundles and slippage along the secondary bundles (Zone III), and iv) final fracture by slippage of the elementary bundles within the secondary bundles (Zone IV).

The considerable improvement in the specific tensile strength of CNTY‐A to that of CNTY‐B originated from the introduction of guide‐roller B, which imparts spin‐line tension that facilitates the assembly of the elementary bundles into secondary bundles. The additional improvement in the specific tensile strength of CNTY‐B to that of CNTY‐M was attributed to a mechanical conditioning process from the assembly of secondary bundles to form larger bundles along with the realignment of the tying elementary bundles. In addition, some of the secondary bundles possess an off‐axis orientation to the relatively well‐oriented secondary bundles along the yarn axis. This can be supported by the fact that the orientation of CNTs mostly within the secondary bundles, as measured by the G peak intensity ratio (*I*_G∥_/*I*_G⊥_) of the parallel direction to the perpendicular direction along the CNTY axis in the polarized Raman spectrum^[^ ^7^ ^]^ (*n* = 5), dramatically improves from 8.6 ± 1.2 (CNTY‐A), 8.9 ± 1.1 (CNTY‐B) to 10.2 ± 1.2 (CNTY‐M) as shown in Figure 3c–e, while there is only a 3% increase in in the effective cross‐sectional area through the mechanical conditioning. Further, the result that the modulus in the elastic zone (Zone I) coincides with the orientation of the secondary bundle shown in Figure 3c–e suggests that the load bearing mechanism in the elastic zone is determined by the secondary bundle. Figure 3f–h shows the extent of the down‐shift of the G’ peak (*n* = 3) on the tensile deformation, which is indicative of the measure of the inter‐tube interaction and hence the inter‐tubular load‐transfer efficiency.^[^ ^25^ ^]^ The secondary bundles oriented slightly off‐the‐yarn axis respond first to the imposed tensile force, showing elastic deformation by realigning to the loading axis (Zone I), followed by an extensional realignment of the misoriented tying secondary bundles and the elementary bundles along the loading axis (Zone II). Subsequent breakage of those tying bundles leads to final rupture through the slippage of the elementary bundles within the last load‐bearing secondary bundles (Zone III and IV). The overall progress of the rupture of CNTYs can be illustrated, as shown in **Figure** **4**. There are considerable differences in the extent of the G’ peak‐down‐shift and the deformable strain range in Zones I and II depending on the samples, as listed in Table S1 (Supporting Information). We observed that the load transfer through bundling behavior is more dominant in plastic region, while orientation behavior has a considerable contribution to the load transfer in elastic region. This observation indicates that the micro‐textural structure development from CNTs to the elementary bundles, then to secondary bundles of various thicknesses and orientations governed by the inter‐bundle interaction energy, varies critically with the process conditions, determining the resulting mechanical performance of the produced CNTYs.

## 3 Conclusion

**Figure 5:**
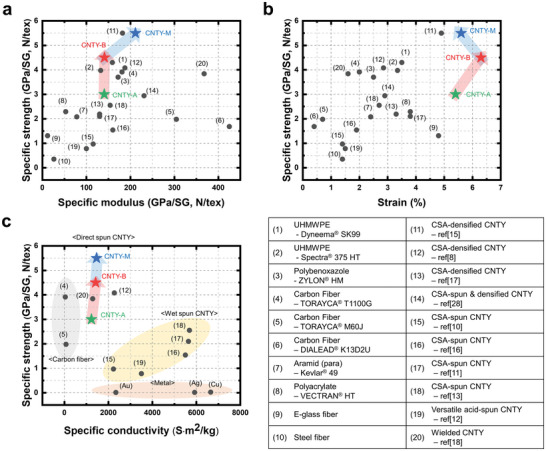
Superior tensile properties of CNTYs relative to other high‐performance fibers. Ashby plots of the present CNTYs (star) in comparison with various high‐performance fibers (circle) in terms of the specific strength versus the a) specific modulus, b) strain, and c) specific electrical conductivity.

The specific mechanical performance of the CNTYs fabricated in this study was outstanding compared to all previously reported CNTYs and most commercialized state‐of‐the‐art high‐performance fibrous materials, as shown in **Figure** **5**. The specific strength and the specific modulus of the as‐spun CNTY‐A itself surpass those of Kevlar aramid fibers, a market‐dominating high‐performance organic fiber. Moreover, the performance of the CNTY‐B is superior to that of Dyneema and Spectra fibers, among the world's strongest and lightest organic fibers available today, and to the carbon fiber Torayca T1100G, the strongest fiber developed thus far. Furthermore, CNTY‐M can be produced in a facile and eco‐friendly manner and possess superior mechanical performance, including a specific tensile strength of 5.5 N tex^−1^ and a specific modulus of 211 N tex^−1^. The strain levels upon failure of all the CNTYs were 5.4–6.3%, which is superior to those of all high‐performance fibers and significant in that the CNTYs sustain large deformation, which allows comparatively easy preforming without breakage of the yarns during composite manufacturing. In addition, the specific electrical conductivity of CNTY‐A, B, and M were 1.23 × 10^3^, 1.42 × 10^3^, and 1.46 × 10^3^ S × m^2^ kg^−1^, respectively. The CNTY sample has shown a high specific conductivity, which are competitive with those of other metals, commercial fiber, and previous reports of CNTY (Figure 5c). Considering even the commercialized materials do not possess both high specific tensile strength and specific electrical conductivity, the result that our optimum CNTY‐M has high mechanical and electrical performances makes our CNTY special.

Based on the concept of structural characteristics of natural fiber, this study highlights a new possibility of producing outstanding CNTYs by bundle engineering in a direct spinning process without post‐treatment. The excellent mechanical performance of these CNTYs was attributed to the well‐developed multiscale micro‐textural structure, i.e., CNTs → elementary bundles → secondary bundles → yarn, which critically depends on the processing conditions of direct spinning. This type of multiscale hierarchical structure, i.e., molecular chains → micro‐fibrils → macro‐fibrils → fibers, is commonly found in natural fibers, such as cellulose and wool fibers, which are quite flexible but strong. Similarly, the superiority of both the tensile strength and the strain at failure of these CNTYs comes from the similarities between the micro‐texture of the CNTYs and natural cellulose fibers. Therefore, for superstrong CNTYs, this study proposes an easy and eco‐friendly bundle engineering method to develop thickened and highly oriented secondary bundles inspired by the micro‐texture of natural fibers.

## 4 Experimental Section

### Materials and the Conditions of the Direct Spinning of DWCNT Yarns

Double‐walled CNTs were synthesized at 1200 °C using a floating catalyst chemical vapor deposition method in a vertical alumina tube reactor with an inner diameter of 85 mm and length of 1800 mm. Ferrocene, as a catalyst precursor, and thiophene, as a promoter, were purchased from Sigma‐Aldrich (South Korea) and used as received. The carbon source, 60 sccm CH_4_ (99.999%), was flowed into a furnace together with 1200 sccm H_2_ (99.999%) and 500 sccm Ar (99.999%). Ferrocene at 80 °C and thiophene at −20 °C were carried by an H_2_ flow into the furnace at rates of 0.5 and 3.6 mg min^−1^, respectively, while keeping the total H_2_ flow rate at 1200 sccm. The grown DWCNTs were spun directly into CNTYs by passing the assembled aerogel‐like web through a water bath, where guide‐roller A was immersed in water. Guide‐roller B was located directly above the water bath, followed by a take‐up roller of which the winding speed varied from 5 to 9 m min^−1^. Mechanical conditioning was then carried out to enhance the development of the micro‐texture of the bundle assembly within the CNTYs. The mechanical conditioning was done by applying cyclic strain of 0.5–2.5% on CNTY only once through tensile tester (TST350, Linkam) at a gauge length of 10 mm to control strain precisely. The strain was applied at the rate of 0.1% s^−1^ and released also at the rate of −0.1% s^−1^ right after strain reached the end point. The CNTY sample taken up directly after running through guide‐roller A only is denoted as CNTY‐A, while that obtained after running through both guide‐rollers A and B is denoted as CNTY‐B. CNTY‐M was obtained by mechanically conditioning the CNTY‐B samples, whereby 2% strain was cyclically applied. (See the Supporting Information for detailed image of fabricating CNTYs.)

### Mechanical Testing and Hierarchical Structure Characterizations of CNTYs

The tensile performance of the CNTYs was tested on a tensile tester (TST350, Linkam) at a gauge length of 10 mm and strain rate of 3 mm min^−1^. The linear density of the given CNTY was determined by measuring the weight of 15 m of CNT yarn. In situ Raman analysis was carried out while the CNTY was strained with a gauge length of 30 mm on a tensile stage set on the sample stage of the Raman spectroscope. The CNT nanostructure was examined by field‐emission transmission electron microscopy (FETEM, JEM‐3000F, JEOL, Japan) and Raman spectroscopy (RAMANplus, Nanophoton) with a 532 nm laser. The purity of the CNTs was determined by thermogravimetric analysis (TGA; SDT‐Q600, TA Instruments) in air. The cross‐ and longitudinal sections of the CNTYs were characterized by field‐emission scanning electron microscopy (FESEM, SUPRA 55VP, Carl Zeiss, Germany) after cutting with a focused ion beam (FIB; Helios 650, FEI).

## Conflict of Interest

The authors declare no conflict of interest.

## Author Contributions

Y.S.C. conceived and designed the experiments. Y.S.C, J.W.L., and Y.J. contributed to the synthesis of CNTYs using the direct spinning method. Y.S.C., J.W.L., and J.W.K. characterized CNTYs and wrote the paper. S.J.Y. and C.R.P. supervised the project. All the authors discussed the results and contributed to the preparation of the paper.

## Supporting information

Supporting Information

## Data Availability

The data that support the findings of this study are available from the corresponding author upon reasonable request.
