# Supplementary material for: Superstrong Carbon Nanotube Yarns by Developing Multiscale Bundle Structures on the Direct Spin‐Line without Post‐Treatment
Source: Adv Sci (Weinh). 2022 Nov 20;10(2):2204250. doi: 10.1002/advs.202204250 (PMC9839856; doi:10.1002/advs.202204250)
Supplement: Supplementary file 1 — Supporting Information [file ADVS-10-2204250-s001.pdf]

ADVANCED SCIENCE
Open Access

Supporting Information

for *Adv. Sci.*, DOI 10.1002/advs.202204250

Superstrong Carbon Nanotube Yarns by Developing Multiscale Bundle Structures on the Direct Spin-Line without Post-Treatment

Young Shik Cho, Jae Won Lee, Jaewook Kim, Yeonsu Jung, Seung Jae Yang and Chong Rae Park**

Supporting information

Superstrong carbon nanotube yarns by developing multiscale bundle structure on the direct spin-line without post-treatment

Young Shik Cho[1], *Jae Won Lee*[1], *Jae Wook Kim*[1], *Seung Jae Yang*[*2] *and Chong Rae Park*[*1]

[1]Department of Materials Science & Engineering and Research Institute of Advanced Materials, Seoul National University, Seoul. 08826, Republic of Korea

[2]Department of Chemistry & Chemical Engineering, Education and Research Center for Smart Energy and Materials, Inha University, Incheon, 22212, Republic of Korea

[*]Corresponding Author.

E-mail address: crpark@snu.ac.kr (C. R. Park), sjyang@inha.ac.kr (S. J. Yang)

Synthesis condition of FCCVD for high strength CNTY-A

Highly crystalline CNTs were synthesized using furnace with a long heating zone, as previously reported in our group[S1]. The input amounts of ferrocene and thiophene were 0.5 and 3.6 mg/min, respectively, as described in the manuscript. Such amounts were increased by 2.8 times and 6.2 times, respectively, compared to the previous report. The atomic ratios of S:C and S:Fe were 0.017 and 15.8, respectively. The increase in the input amount of ferrocene was to increase the linear density of CNTY from 0.05 tex to 0.11 tex. The increase in input ratio of thiophene was higher than that ferrocene to increase the stabilization efficiency of synthesized iron nanoparticles through reaction with sulfur[S2]. Because the overall flow was slowed due to the large diameter of furnace from Bernoulli's principle, iron nanoparticles collided with each other and increased to stabilize. However, increase in reaction between iron nanoparticle and sulfur resulted in maintenance of the catalyst activity by stabilizing the nanoparticle without size growth. Furthermore, the atmosphere in the furnace was created by adding Ar to H_2. Addition of Ar, which does not participate in any reaction, led to increase in flow rate of total gas with the improvement in reaction frequency of iron and sulfur, showing similar effect to deep injection method[S3]. As a result, owing to improved synthesis efficiency of CNT from catalyst, it was possible to fabricate a DWCNT-based CNTY with enhanced physical & mechanical properties.

Estimation of aspect ratio of CNT from CNTY-M

Considering that CNTY-M is almost completely densified and consists of huge secondary bundles, the aspect ratio of CNT can be predicted by comparing with the CSA-spun CNTYs, which are known to be made up of almost one secondary bundle. According to previous report[S4], the strength of CNTY is proportional to (aspect ratio)$^{0.9}$. CNTY-M has a density of 0.36g/cm^3 and a specific strength of 5.5 N/tex, stating that the strength of CNTY-M is about 1.94 GPa. Meanwhile, the strength of the CSA-spun CNTY in the previous report[S5] is 4.2 GPa and the aspect ratio of the CNT used is 6700. Based on the simple calculation, the aspect ratio of our DWCNT is estimated to be approximately 2800.

Fabrication of CNTYs

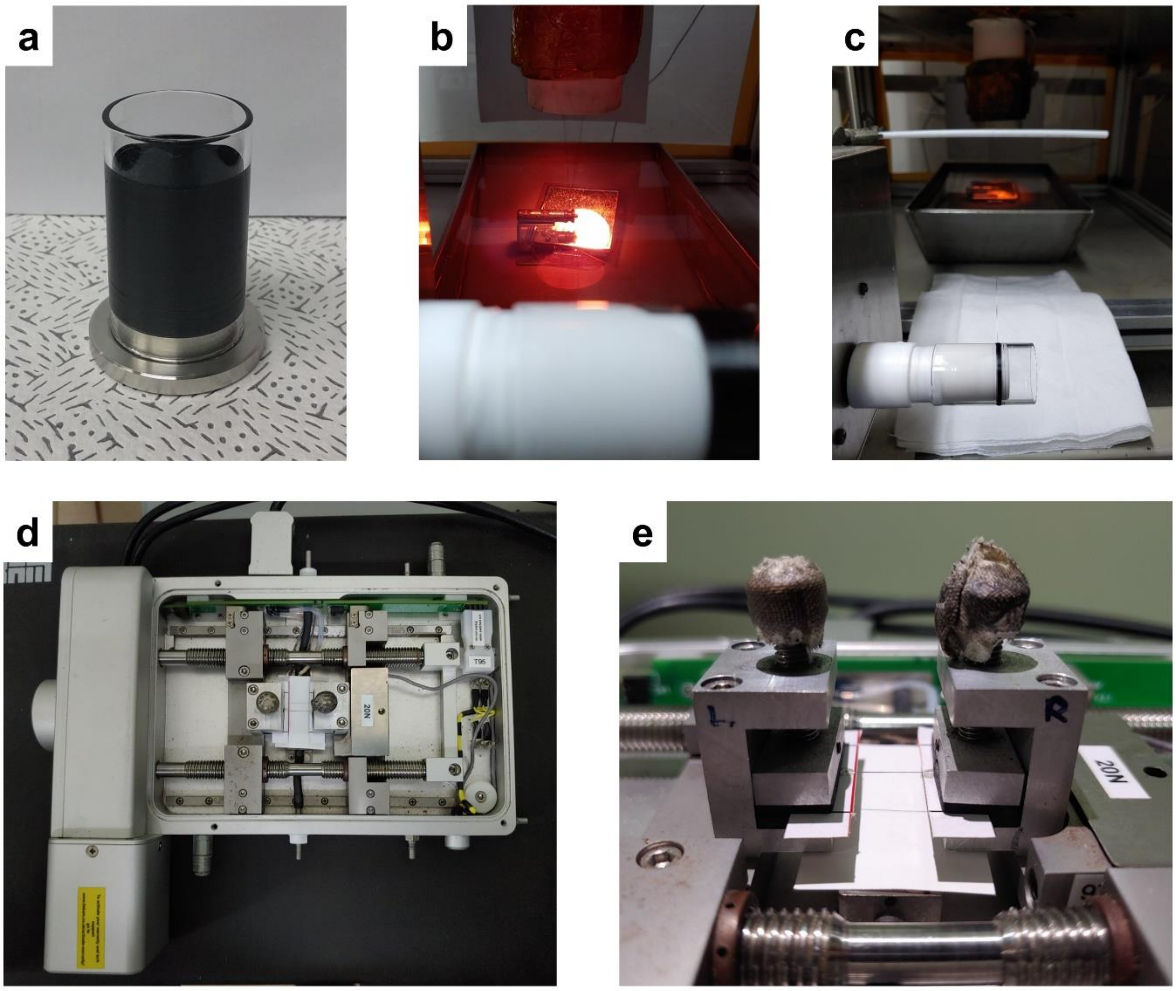

Figure S1. Synthesis images of CNTYs: (a) fabricated CNTY, direct spinning process (b) without and (c) with guide roller B, and (d, e) mechanical conditioning process through tensile tester.

Structure of individual DWCNT and its assembly

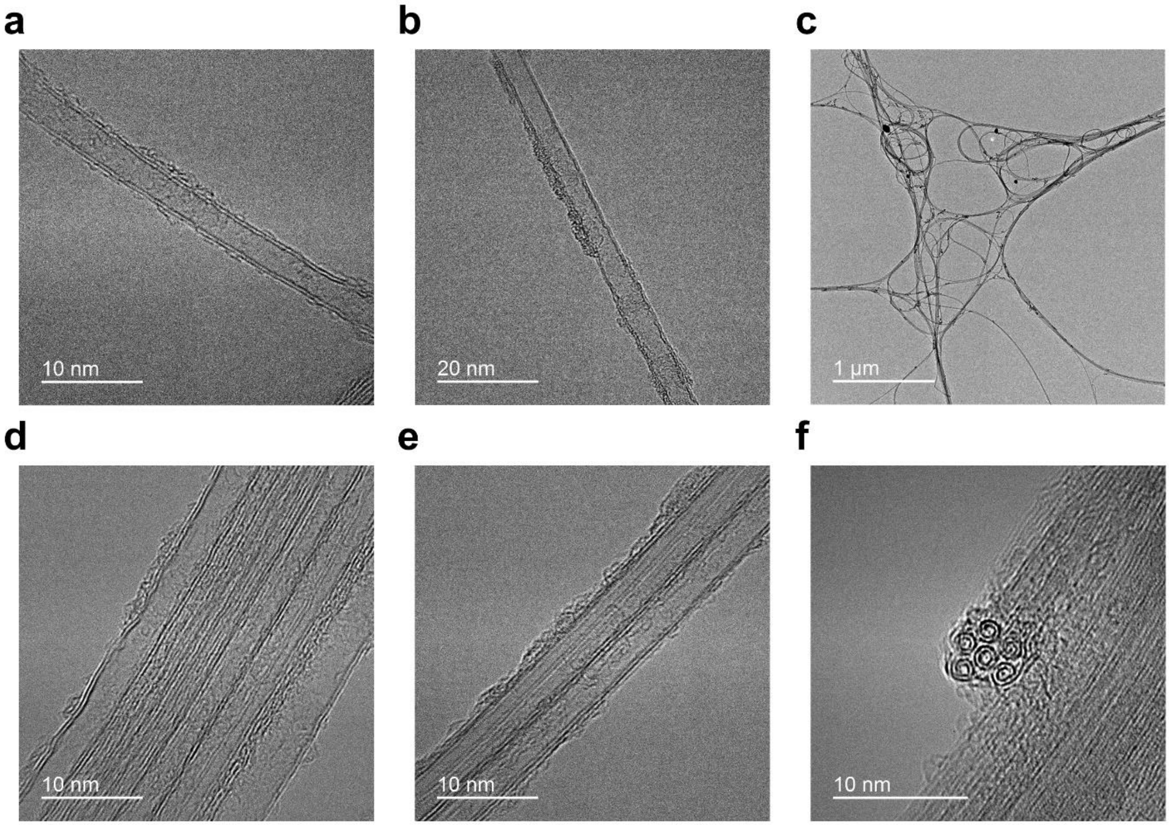

Figure S2. TEM images of (a, b) as-synthesized individual DWCNT, (c) network structure of DWCNTs, (d, e) elementary bundles of DWCNT with (f) cross-sectional view.

Homogeneity of CNTYs

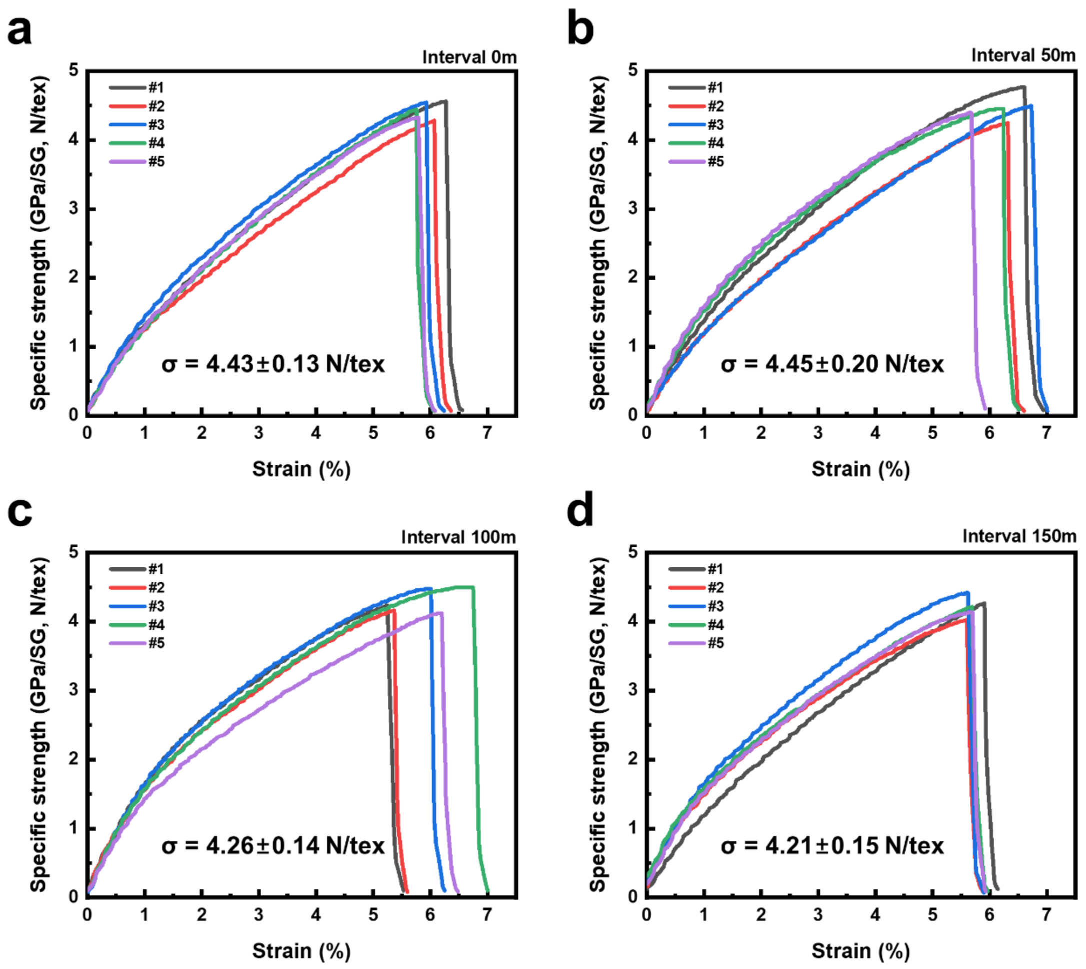

Figure S3. Homogeneity of CNTY: The stress-strain curve of CNTY-B at interval of (a) 0m, (b) 50m, (c) 100m, and (d) 150m ($n = 5$).

The homogeneity of CNT yarns is important when synthesizing yarns in over hundreds of meter length scale. Therefore, physical properties analysis of CNTY-B, for example, measured at every 50m interval, was performed. The CNT yarns were maintained almost uniformly as shown in the stress-strain curve of Fig. S3. The specific strength of CNTY-B in each 50m interval was 4.43, 4.45, 4.26, 4.21 N/tex, respectively, proving the uniformity of our CNTY.

Mechanical optimization of CNTY

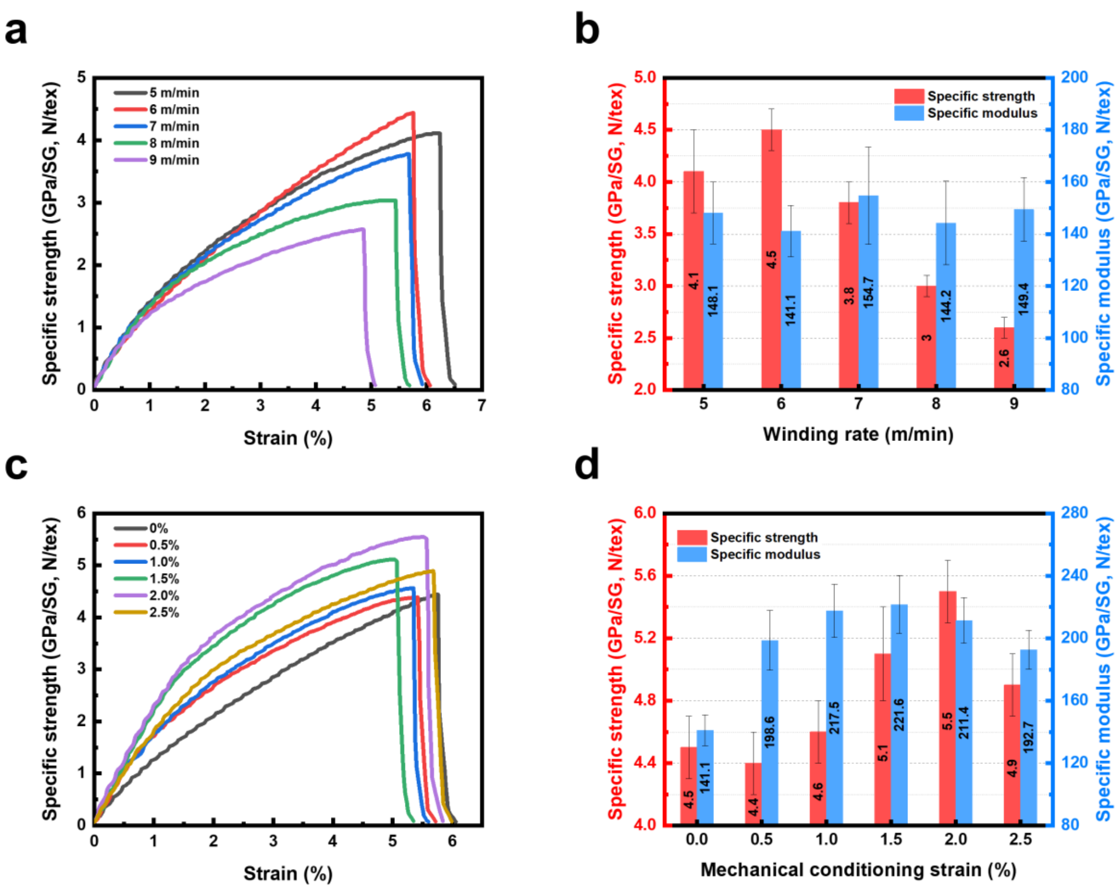

Figure S4. Optimization of CNTY: (a, c) the stress-strain curve and (b, d) mechanical properties of CNTY-B according to winding rate (top, n = 10) and CNTY-M according to cyclic strain (bottom, n = 10).

When the winding rate is 6 m/min in the presence of the guide roller, the specific strength shows the highest value due to strain-derived self-assembly of CNT bundles (Figure S1a-b). As the rate increased, the specific strength decreased, which matches the self-assembly behavior of CNT bundle. This is presumed to be due to excessive drawing to the extent that it corresponds to Zone III as the winding rate increases. In the case of mechanical conditioning, when about 2% of cyclic strain is applied, the specific strength is at its highest level (Figure S1c-d). Likewise, strain caused bundle dense packing before the optimal point, but then excessive drawing led to a decrease in the mechanical properties of CNTY.

Assembly state of hierarchical bundles in CNTYs with verification of elementary bundle

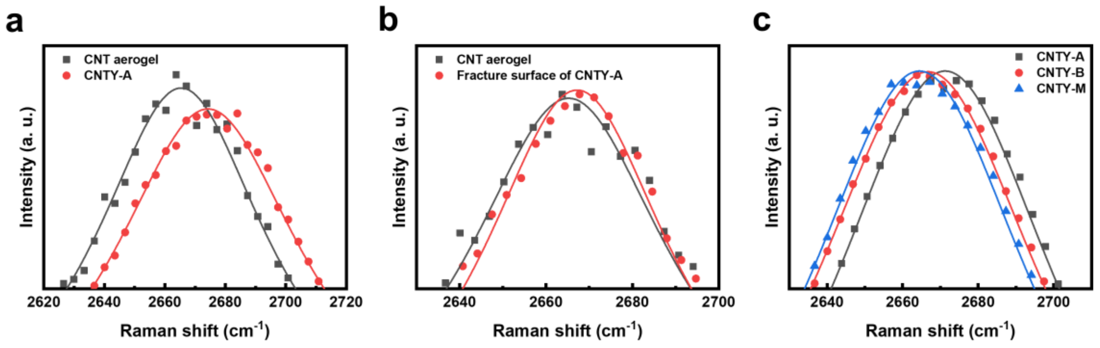

Figure S5. Comparison of Raman spectra of the G' peak (symbol) that is fitted using a Lorentzian function (solid line): (a) CNT aerogel & CNTY-A, (b) CNT aerogel & fracture surface of CNTY-A, and (c) DWCNT yarns (n = 5).

Fig. S5a shows G' Raman band of CNT aerogel and CNTY-A with the fitted line of Lorentzian function. The peak position of CNT aerogel was 2665.4 cm^{-1} (G' band) while that of CNTY-A was 2674.3 cm^{-1} (G' band), showing G' band has upshifted 8.9 cm^{-1}. Because interaction of CNTs in nanoscaled elementary bundle is far higher than that of CNTs between elementary bundles in secondary bundle and CNT aerogel is mainly composed of elementary bundles rather than secondary bundle, the peak position of CNT aerogel showed lower value. As CNT aerogels transforms into CNTY and elementary bundles aggregate into secondary bundles, the addition of interaction between elementary bundles on interaction between CNTs leads to decrease in average interaction of CNTs with upshift of Raman bands.

In addition to the above results, the elementary bundle with a certain diameter (30 nm) appears as shown in the fracture surface of Fig. 2b-c. If the elementary bundle does not maintain its structure, the size of the bundle at the end point of the fracture surface would not have been constant. In other words, because the elementary bundle maintained its structure, slippage occurred at the interface between the elementary bundles, which is the weakest point in the secondary bundle. Furthermore, the G' band of the fracture surface of CNTY-A shows that the peak position appears at 2667.2 cm^{-1}, decreasing from CNTY state and becoming similar to elementary bundles (Fig. S5b). As the secondary bundles within the CNTY fails and the relatively weak interaction disappears, the interaction between the CNTs in the elementary bundle formed a G' band. Therefore, such results verify that the elementary

bundle maintains its structure and plays a role even after the CNTY is formed.

Detailed development of hierarchical micro-texture of bundles in CNTYs

Table S1. Strain and G' peak down-shift of CNTYs in bundling region (Zone I & II)

	Zone I		Zone II		Total	
	Strain (%)	G' down shift (cm^{-1})	Strain (%)	G' down shift (cm^{-1})	Strain (%)	G' down shift (cm^{-1})
CNTY-A	0.70	-10.13	1.05	-7.42	1.75	-17.55
CNTY-B	0.75	-11.10	2.15	-13.39	2.90	-24.49
CNTY-M	1.05	-16.50	1.70	-9.91	2.75	-26.41

Comparing the DWCNT yarns based on the above analysis, significant changes were found in Zones I and II. First, when comparing the *in-situ* Raman analysis of CNTY-B (Fig. 3g) and CNTY-A (Fig. 3f), not only did the strain in the bundling region (Zone I+II) increase from 1.75% to 2.90%, but the G' downshift also enhanced from 17.55 cm^{-1} to 24.49 cm^{-1}. Such change was mainly observed in Zone II, and as summarized in Table S1, strain and G' down shift increased by 1.1% and 5.97 cm^{-1}, respectively. Considering that the main load transferring element of Zone II is an elementary bundle, the result indicates that the engineering carried out in the direct spinning process to produce the CNTY-B is based on the self-assembly of the elementary bundle.

On the other hand, when comparing *in-situ* Raman analysis of CNTY-M (Fig. 3h) and CNTY-B (Fig. 3g), As the mechanical conditioning progressed, though the overall strain of the bundling region was slightly decreased, the G' downshift was rather increased. In particular, significant changes were observed in the strain and G' down shift ratios occupied by Zones I and II in the bundling region, respectively. As the train of, Zone I widened from 0.75% to 1.05% and the G' downshift of Zone I increased from 11.10 cm^{-1} to 16.50 cm^{-1}, the main load transferring region changed from Zone II to I. In CNTY-M, the specific strength increased as the secondary bundle additionally contributed to the load transfer. The result indicates that the mechanical conditioning process performs secondary bundle-based

engineering as in polymer fibers. The suggested mechanism states that this assembly behavior engineering of CNT was the key to improving the specific strength of CNTY.

Reference

[S1] J. Lee, D. M. Lee, Y. Jung, J. Park, H. S. Lee, Y. K. Kim, C. R. Park, H. S. Jeong, S. M. Kim, *Nat. Commun.* **2019**, 10, 2962.

[S2] M. S. Motta, A. Moisala, I. A. Kinloch, A. H. Windle, *J. Nanosci. Nanotechnol.* **2008**, 8, 2442.

[S3] S.-H. Lee, J. Park, J. H. Park, D.-M. Lee, A. Lee, S. Y. Moon, S. Y. Lee, H. S. Jeong, S. M. Kim, *Carbon* **2021**, 173, 901.

[S4] D. E. Tsentalovich, R. J. Headrick, F. Mirri, J. Hao, N. Behabtu, C. C. Young, M. Pasquali, *ACS Appl. Mater. Interfaces* **2017**, 9, 36189.

[S5] L. W. Taylor, O. S. Dewey, R. J. Headrick, N. Komatsu, N. M. Peraca, G. Wehmeyer, J. Kono, M. Pasquali, *Carbon* **2021**, 171, 689.
